# Machine-Learning Based Channel Quality and Stability Estimation for Stream-Based Multichannel Wireless Sensor Networks

**DOI:** 10.3390/s16091476

**Published:** 2016-09-12

**Authors:** Waqas Rehan, Stefan Fischer, Maaz Rehan

**Affiliations:** 1 Institute of Telematics (ITM), University of Luebeck, 23562 Luebeck, Germany; fischer@itm.uni-luebeck.de; 2 COMSATS Institute of Information Technology (CIIT), Quaid Avenue, 47040 Wah Cantt, Pakistan; maazrehan@ciitwah.edu.pk

**Keywords:** multichannel, multi-radio, channel quality prediction, wireless sensor networks, machine learning

## Abstract

Wireless sensor networks (WSNs) have become more and more diversified and are today able to also support high data rate applications, such as multimedia. In this case, per-packet channel handshaking/switching may result in inducing additional overheads, such as energy consumption, delays and, therefore, data loss. One of the solutions is to perform stream-based channel allocation where channel handshaking is performed once before transmitting the whole data stream. Deciding stream-based channel allocation is more critical in case of multichannel WSNs where channels of different quality/stability are available and the wish for high performance requires sensor nodes to switch to the best among the available channels. In this work, we will focus on devising mechanisms that perform channel quality/stability estimation in order to improve the accommodation of stream-based communication in multichannel wireless sensor networks. For performing channel quality assessment, we have formulated a composite metric, which we call channel rank measurement (*CRM*), that can demarcate channels into good, intermediate and bad quality on the basis of the standard deviation of the received signal strength indicator (RSSI) and the average of the link quality indicator (LQI) of the received packets. CRM is then used to generate a data set for training a supervised machine learning-based algorithm (which we call Normal Equation based Channel quality prediction (NEC) algorithm) in such a way that it may perform instantaneous channel rank estimation of any channel. Subsequently, two robust extensions of the NEC algorithm are proposed (which we call Normal Equation based Weighted Moving Average Channel quality prediction (NEWMAC) algorithm and Normal Equation based Aggregate Maturity Criteria with Beta Tracking based Channel weight prediction (NEAMCBTC) algorithm), that can perform channel quality estimation on the basis of both current and past values of channel rank estimation. In the end, simulations are made using MATLAB, and the results show that the Extended version of NEAMCBTC algorithm (Ext-NEAMCBTC) outperforms the compared techniques in terms of channel quality and stability assessment. It also minimizes channel switching overheads (in terms of switching delays and energy consumption) for accommodating stream-based communication in multichannel WSNs.

## 1. Introduction

Wireless sensor networks (WSNs) consist of tiny devices, which have limited energy, memory, sensing/processing unit and transmission capability [1]. Such networks often also have the capacity of self-organization in ad hoc mode [2,3]. Traditionally, energy efficiency has always been a key issue in WSNs, whereas throughput, fairness and delay were not so much the focus of research [4]. However, technological advancement has shifted this focus and enabled sensor nodes to transmit large volumes of scalar environmental data, e.g., concerning temperature, humidity or pressure. Meanwhile, camera and microphone-enabled sensor nodes have emerged; networks build from such nodes are called wireless multimedia sensor networks (WMSNs) [5,6] and have the ability to transmit very large volumes of delay-sensitive multimedia data to sink nodes. Additionally, a variety of multipath and multichannel approaches have been proposed by researchers whose purpose is to further improve the performance of WSNs.

**Figure 1:**
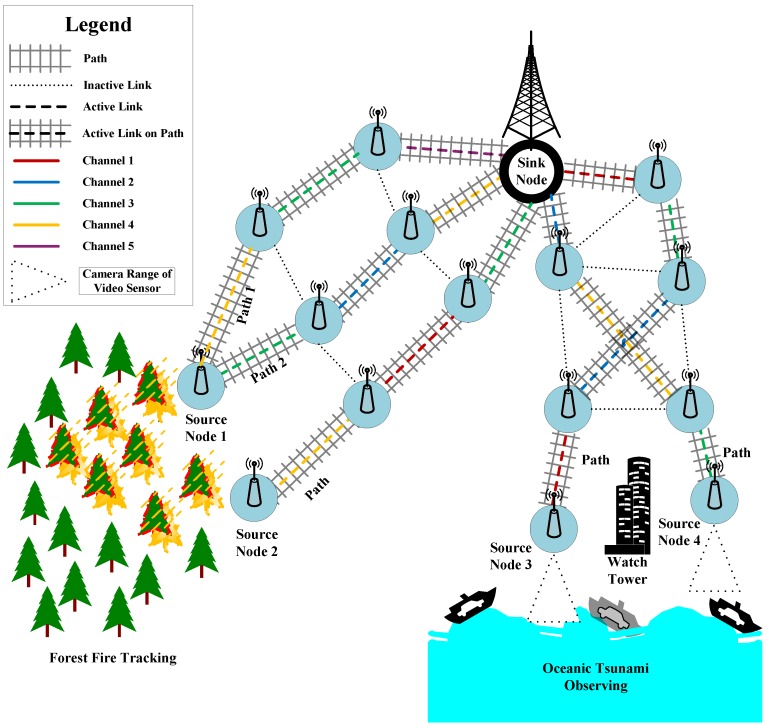
Surveillance using a multichannel/multipath WSN.

Figure 1 shows a sample scenario using multiple paths and channels. Source Node 1 and the sink node are connected by two paths, whereas the remaining sources can communicate with the sink using only a single path. Any two neighbor nodes on a path have an active link between them, which resides on the best among all available channels.

Unlike sensor nodes in conventional networks, nodes in multichannel sensor networks additionally have to deal with a potentially large number of channels for data routing, i.e., they have to decide which channel to use for transmission; the better the choice the better the overall system performance. Therefore, an appropriate channel quality/stability assessment is needed. Since the behavior of wireless channels is probabilistic in nature (which makes channel quality assessment a recurrent task), any instantaneous channel quality assessment cannot, by nature, provide an adequate measure of the channels’ health. In [7], the authors have realized that combining current and past channel quality assessments at the receiver may help to predict the channel quality indicator (CQI), which may guide the transmitter to adapt the transmission parameters accordingly and improve the performance of the wireless communication systems.

A large number of multichannel protocols for WSNs uses single radio per node [8,9,10,11,12]. If transmitting and receiving frequencies of sensor nodes are different, then such sensor networks may suffer from channel switching delays and additional power consumption [12]. When the data rate is very high, frequent channel switching may result in data loss [12], which may adversely affect the system performance. Therefore, for accommodating multimedia traffic, it is more efficient and cost-effective to aim for a stream-based channel assignment rather than to do this on a per-packet base [13,14]. However, channel behavior is not deterministic in nature; therefore, reserving a channel for a whole data stream requires knowledge about average channel response, which may be assessed in advance through channel quality and stability assessment. To the best of our knowledge, there are no multichannel protocols in WSNs that embody any mechanism of channel quality and stability assessment for supporting stream-based channel allocation in multichannel WSNs.

The quality of a channel in a neighborhood can be assessed on the basis of link quality in that vicinity. In [15], a link quality-based channel selection approach is proposed, which ranks channels into different categories and selects good quality channels for improving system performance. Moreover, for measuring link quality, different authors have introduced distinct metrics, such as [16,17,18,19]. The author in [20] has realized that good, intermediate and bad links can be discriminated on CC2420 using the standard deviation of RSSI ( std(RSSI) ) and the average of link quality indicator (LQI) ( avg(LQI) ), as shown in Table 1. The advantage of using std(RSSI) and avg(LQI) is that they may measure performance and central tendency, respectively, of link quality in a better manner. On the other hand, the average of RSSI ( avg(RSSI) ) and the standard deviation of LQI ( std(LQI)) are not good estimators of link quality because they may cause overlap of bad with intermediate quality links and intermediate with good quality links, respectively [20]. Since various link quality metrics have their own limitations [21], therefore no consensus has been developed among the research community for the most suitable link quality metric [17]. Consequently, a hybrid metric may ultimately be used for accurately accessing link quality [21].

Most of the multichannel protocols in WSNs do not consider any scheme of channel quality assessment before channel assignment. For example, tree Construction and Channel Allocation algorithm (CCA) [22], Iterative Channel Adjustment Data Aggregation Routing algorithm (ICADAR) [23], Lagrangean Relaxation algorithm (LGR) [24,25] do not employ any mechanism to differentiate between good and bad quality channels. On the other hand, there are some multichannel protocols in WSNs that employ different mechanisms for measuring channel quality, such as Efficient Multichannel MAC protocol (EM-MAC) [26], Decentralized Optimization for Multichannel Random Access (DOMRA) [27], Multi-radio Multi-channel Opportunistic Cooperative Routing algorithm (MMOCR) [28], Distributed Routing and Channel Selection scheme (DRCS) [12], Regret Matching based Channel Assignment algorithm (RMCA) [29,30]. However, to the best of our knowledge, there is no multichannel protocol in WSNs that can make channel quality and stability assessment on the basis of both current and past channel quality data.

In this paper, we focus on accomplishing channel quality and stability assessment for accommodating stream-based communication in WSNs. In this context, the main contributions of our work can be summarized as follows:

- Formulating a composite channel rank measurement ( CRM ) metric that is used to create a dataset for training the normal-equation-based predictor.
- Employing a normal-equation-based supervised machine learning algorithm (NEC algorithm) and training it using a channel quality-based generated dataset in such a way that it performs channel rank estimation $\left(CRE_{t}^{i}\right)$ of any channel *i* based on only instantaneous values of $std(RSSI_{t}^{i})$ and $avg(LQI_{t}^{i})$ of received packets.
- Extending the NEC algorithm by devising the NEWMAC algorithm, which employs a weighted moving average-based criterion for predicting the final channel rank estimation $\left(\phi_{t,NEWMAC}^{i}\right)$ of any channel *i* based on both past and current values of channel quality prediction.
- Devising the NEAMCBTC algorithm as an extension of NEC algorithm, which employs an adaptive weighting procedure by considering past and current channel quality predictions for estimating the final channel rank estimation $\left(\phi_{t,NEAMCBTC}^{i}\right)$ of any channel *i* and also promptly tracking channel quality degradations/upgradations. For more robustness, devising an extended version, entitled as Ext-NEAMCBTC algorithm that may perform both channel quality and stability assessment as a composite task.

The rest of the paper is structured as follows. In Section 2, we discuss the related work already mentioned above in a more detailed manner and draw our conclusions for the motivation of our research. Section 3 explains the underlying system model. Section 4 discusses the channel quality assessment metric. In Section 5, we elaborate the proposed supervised machine learning-based algorithms along with their problem statements. Section 6 presents and discusses the detailed performance evaluations of those algorithms along with their pros and cons. Section 7 sums up the overall presented work and draws conclusions.

## 2. Related Work and Motivation

The objective of this paper is to propose a robust multichannel algorithm that may perform both channel quality and stability assessment to support stream-based communication in WSNs. For this purpose, we have studied a large number of multichannel techniques in WSNs and found a limited number of protocols that embody some channel quality assessment mechanism for achieving high performance.

Tang et al. [26] have proposed the EM-MAC algorithm where the channel quality assessment criterion is maintained through the Clear Channel Assessment (CCA) technique. When a congested/interfered channel is encountered, it is marked as blacklisted and avoided till the end of the current session. In this way, bad quality channels are differentiated from good ones. Similarly, Jingrong et al. [28] have introduced a new channel quality-based metric called channel interference strength (CIS), which is based on a composite metric of power received $P_{r}$ and Signal-to-Interference-plus-Noise Ratio (SINR). It allows sensor nodes to select the channel with the smallest CIS for future communication. The CIS-based metric does not consider previous channel quality and focus on instantaneous measures.

Khan et al. [29] have proposed a game theory-based multichannel protocol for WMSNs where a channel is selected by a cluster head for the next round when that channel is not selected by its neighboring clusters in the previous round. Otherwise, avg(LQI) is used as a metric for channel selection; however, the approach does not outline how it does that, and it does not consider past channel quality in future channel quality assessment. Similarly, Pal et al. [12] have utilized node energy and an expected number of transmissions (ETX)-based channel quality assessment metric for performing future communication. Although this ETX-based metric is reliable, it may require probing packets and, consequently, is costly to carry out. Moreover, it only considers instantaneous channel quality and does not regard past channel quality and stability assessment for measuring the final channel quality estimation. Likewise, Yu et al. [30] have presented a multichannel protocol where each node maintains a utility function and a past information-based performance matrix that helps to predict future network topology/flows and actions of neighbor nodes. Subsequently, channels are assigned accordingly. The protocol makes future channel assignment on the basis of predictions based on past knowledge only and does not consider current channel quality assessment.

Finally, to the best of our knowledge, we can conclude the related work as outlined in the Table 2, where the summarized results clearly show that the multichannel protocols in WSNs are either considering as instantaneous channel quality assessment or selecting the final channel on the basis of some past knowledge. Therefore, we can conclude that no multichannel protocol is available in WSNs that considers both current and past channel quality estimation to predict final channel rank estimation in WSNs.

In addition to the above discussion, we have noted earlier that std(RSSI) and avg(LQI) of received packets may discriminate links into different categories [20], as shown in detail in Table 1. Since each link in the neighborhood of a node may use a specific frequency channel for transmission as shown in Figure 1, the quality of these links in the neighborhood of a node may determine the overall quality of a channel in this neighborhood. Consequently we have formulated the CRM metric that is used to train a normal equation-based predictor for executing channel rank estimation $\left(CRE_{t}^{i}\right)$ of any channel *i* at instant *t* using $std(RSSI_{t}^{i})$ and $avg(LQI_{t}^{i})$ of received packets.

Our second observation from the literature review is that there is no multichannel protocol in WSNs that employs channel quality and stability assessment, using present and past channel knowledge to accommodate stream-based applications. Two of the three algorithms we present in this paper, namely NEWMAC and NEAMCBTC, are closing this gap. We also believe that this is the first work that employs a normal equation-based supervised machine learning algorithm for channel quality approximation in multichannel WSNs.

## 3. System Model

We model a WMSN as a directed graph G(S,E) where the set of vertices *V* represent *N* multimedia-enabled sensor nodes, i.e., $V=\{n_{i}|i=1,2,3,\cdots ,N\}$ . The sensor nodes are randomly distributed in the sensing field and may be static or dynamic in nature. There is a bidirectional edge e∈E between any two neighboring vertices $n_{i}$ and $n_{j}$ , which may enable them to perform channel negotiation with each other.

The physical layer model allows each sensor node to compute $std(RSSI_{t}^{i})$ and $avg(LQI_{t}^{i})$ of received packets on any channel *i* at instant *t*. Afterwards, machine learning-based technology is employed to estimate the quality of the corresponding channel.

The MAC layer model allows each sensor node to sense available channels in the neighborhood and perform channel negotiation with the preferred neighbor node on a path in a manner that the available channel of highest quality is negotiated first, then the one with the second-highest quality, and so on. Once channel handshaking has been performed and the best channel is agreed on, both sender and receiver jump to the desired channel for performing stream-based communication and stay there till the data stream ends. All channels are of equal bandwidth and orthogonal in nature. Additionally, it is assumed that all channels are not jammed or degraded simultaneously, and therefore, some channels of good quality are always available for performing stream-based communication.

For simplicity, we assume that the quality of all links on a particular frequency channel in a neighborhood is the same and may reflect channel quality in the corresponding locality. Otherwise, each sensor node may have to record the quality of all available channels for each link in a neighborhood separately, which may increase system complexity accordingly.

## 4. Channel Rank Measurement

We noted earlier that std(RSSI) and avg(LQI) of received packets are useful parameters to describe the link quality [20] and, thus, also the channel quality. In this section, we will discuss a mathematical formulation of our channel rank measurement CRM metric, which is used to create the dataset for training our normal-equation-based channel quality predictor.

On the basis of Table 1, it is clear that the values of std(RSSI) and avg(LQI) have different ranges and spreads; therefore, for getting the benefits of both worlds, we first have to bring these channel quality metrics into a common scale for calculating the channel quality measurement ( CQM ) metric. In this way, the final impact of std(RSSI) and avg(LQI) is approximately equalized, and therefore, a clear boundary can be drawn between good, intermediate and bad quality links. The CQM metric is calculated as:

(1) CQM=[scale(LQI)+scale(RSSI)]

where

(2) $$scale\left(LQI\right)=\left[\frac{avg(LQI)-min(LQI)}{\gamma }\right]$$

(3) scale(RSSI)=[σ−std(RSSI)]

where, following [20,31,32], min(LQI)=50 . We choose the values of the scaling parameters σ=15 and γ=4.0 in order to bring std(RSSI) and avg(LQI) into a common scale.

**Figure 2:**
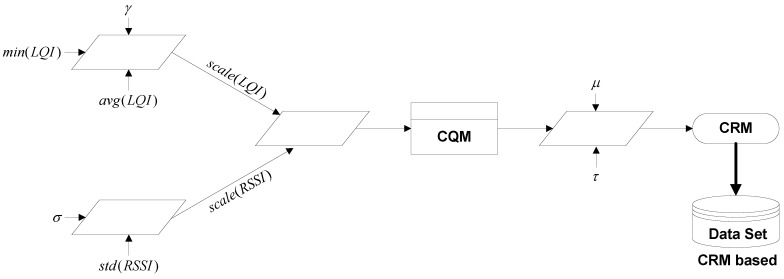
Diagrammatic representation of channel rank measurement (CRM) metric-based training dataset generation.

Our CRM , then, is given by:

(4) $$CRM=\left[\frac{CQM\times \tau }{\mu }\right]$$

where *τ* = 3.5 and *μ* = 100 are adjustment parameters that constrain the values of the CRM-based channel quality training dataset in the range [0, 1] as shown in Table 3. Thus, channel rank estimation (CRE) will fall into the unit interval and, subsequently, channel manipulation of all quality levels is in the range [0, 1]. The detailed representation of CRM based metric is shown in Figure 2, while channel classification into different categories is explained in Table 3. In the next section, we will explain how the CRM -based dataset may be used to perform CRE .

## 5. Supervised Machine Learning-Based Prediction Algorithms

Since each sensor node has limited energy, it is important to employ cost-effective algorithms in WSNs. For this purpose, a dataset is generated on the basis of calculations made in Section 4 and is used to train our basic normal-equation-based machine learning algorithm called normal equation-based channel quality prediction (NEC). Afterwards, we propose two more sophisticated extensions of the basic NEC algorithm, namely normal equation-based weighted moving average channel quality prediction algorithm (NEWMAC) and normal equation-based aggregate maturity criteria with beta tracking-based channel weight prediction algorithm (NEAMCBTC), which consider both instantaneous and past values of channel quality for making final channel quality prediction. We will discuss the mathematical background of normal equation-based prediction in Section 5.1 and the proposed algorithms in Section 5.2, Section 5.3 and Section 5.4, and we also elaborate on their pros and cons.

### 5.1. Normal Equation-Based Prediction

The normal equation-based prediction is more feasible and cost effective when the number of features is small. Since, in this work, we are considering two features i.e., $std(RSSI_{t}^{i})$ and $avg(LQI_{t}^{i})$ , it is more cost effective to use normal equation-based channel quality prediction rather than employing a gradient descent algorithm for making channel quality assessment.

Consider an over-determined system where m is the number of training examples (corresponding to m linear equations) and n is the number of features (corresponding to n unknown coefficients, i.e., $\theta_{1},\theta_{2},\theta_{3},\ldots ,\theta_{n}$ ) with m>n , then the system can be expressed as [33]:

(5) $$\sum_{j=1}^{n}X_{ij}\theta_{j}=y_{i},\;\;where\;i=1,2,3,\ldots ,m$$

In the matrix form [33], we can write:

(6) Xθ=y

where **X** is the feature matrix, ***θ*** is the learning coefficients vector and **y** is the output vector given by:

$$X=\left[\begin{array}{ccccc} x_{11} & x_{12} & x_{13} & \cdots & x_{1n}\\ x_{21} & x_{22} & x_{23} & \cdots & x_{2n}\\ \vdots & \vdots & \vdots & \ddots & \vdots \\ x_{m1} & x_{m2} & x_{m3} & \cdots & x_{mn} \end{array}\right],\;\theta =\left[\begin{array}{c} \theta_{1}\\ \theta_{2}\\ \vdots \\ \theta_{n} \end{array}\right],\;y=\left[\begin{array}{c} y_{1}\\ y_{2}\\ \vdots \\ y_{m} \end{array}\right]$$

Now, by solving the quadratic minimization problem, *θ* is given by [33]:

(7) $$\hat{\theta }=argmin_{\theta }\emptyset \left(\theta \right)$$

Here, ∅ is the objective function and is given by [33]:

(8)∅(θ)=∑i=1m|yi−∑j=1nXijθj|2=||y−Xθ||2(9)=(y−Xθ)T(y−Xθ)=yTy−θTXTy−yTXθ+θTXTXθ(10)=yTy−2θTXTy+θTXTXθ,(asθTXTy=yTXθ)

Taking the derivative of the above equation with respect to ***θ*** and equating it to zero, we get the normal equation [33] as shown below:

(11) $$\theta ={\left(X^{T}X\right)}^{-1}+X^{T}y$$

The above equation clearly indicates that $X^{T}X$ is an [n×n] matrix. Therefore, the cost of inverting this matrix is $O(n^{3})$ . However, it is affordable in our case, because the number of features are only two, i.e., std(RSSI) and avg(LQI) . The training of the system with the dataset is carried out only once, at the start of the system execution phase, and the learning coefficients vector ***θ*** is calculated using Equation (11). Since we are dealing with only two features, therefore ***θ*** and **y** would be [(2+1)×1] and [m×1] vectors, respectively. Moreover **X** and $X^{T}$ would be [m×(2+1)] and [(2+1)×m] matrices, respectively as given below:

$$X=\left[\begin{array}{ccc} 1 & x_{11} & x_{12}\\ 1 & x_{21} & x_{22}\\ \vdots & \vdots & \vdots \\ 1 & x_{m1} & x_{m2} \end{array}\right],\;\theta =\left[\begin{array}{c} \theta_{0}\\ \theta_{1}\\ \theta_{2} \end{array}\right],\;y=\left[\begin{array}{c} y_{1}\\ y_{2}\\ \vdots \\ y_{m} \end{array}\right]$$

### 5.2. Normal Equation-Based Channel Quality Prediction Algorithm

**Figure 3:**
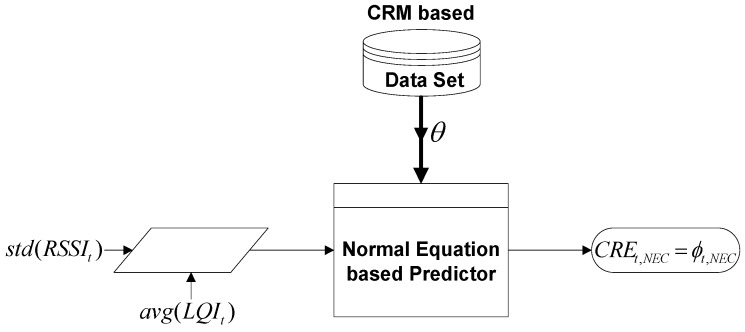
Data flow diagram of the NECalgorithm.

The NEC algorithm can perform channel rank estimation $CRE_{t}^{i}$ of any channel i on the basis of current values of $std(RSSI_{t}^{i})$ and $avg(LQI_{t}^{i})$ of received packets on the corresponding channel, as shown in Figure 3. Since it employs two input features, it is more efficient to use the normal equation-based machine learning algorithm for solving ***θ***. Henceforth, the hypothesis function for estimating $CRE_{t}^{i}$ is as follows:

(12) $$h_{\theta }\left(X\right)=\theta_{o}X_{o}+\theta_{1}X_{1}+\theta_{2}X_{2}$$

where $h_{\theta }\left(X\right)=\;CRE_{t,NEC}^{i}\;,\;X_{o}=1,\;X_{1}=std\left(RSSI_{t}^{i}\right)$ and $X_{2}=avg\left(LQI_{t}^{i}\right)$ . Moreover, the leaning coefficients $\theta_{o},\theta_{1}$ and $\theta_{2}$ are measured on the basis of available dataset.

#### 5.2.1. Problem Definition

Let $\phi_{t,NEC}^{i}$ be the measure of channel quality, i.e., ${\left(CRE\right)}_{t,NEC}^{i}$ of any channel *i* at the instant *t*. Let *C* denote a set of all channels in the neighborhood and *Z* denote a set of all channels, except *i*, i.e., *C* = *Z* + *i*. Then, the objective function *F* is to select a channel *i* at instant *t* that exhibits the maximum quality:

(13) $$\text{Maximize}:\;\;F(\phi_{t,NEC}^{i}),\;\;\;\;\;\;\;i=1,2,3\ldots C$$

(14) $$\text{Subject}\;\text{to}:\;{\left(CRE\right)}_{t,NEC}^{i}\geq {\left(CRE\right)}_{t,NEC}^{Z}\;,\;C=\{i+Z/i\notin Z\}$$

where:

$$0\leq (\phi_{t,NEC}^{i},{\left(CRE\right)}_{t,NEC}^{i})\leq 1.0$$

The above constraint (Equation (14)) elaborates that if any channel i has the highest channel rank estimation ${\left(CRE\right)}_{t,NEC}^{i}$ at instant *t*, then it would be more suitable to accommodate stream-based data communication. Since the NEC algorithm accesses multichannel quality on the basis of instantaneous channel knowledge only; therefore, it may not be suitable to address stream-based communication that requires average channel knowledge at a particular epoch for accommodating the whole data stream afterwards.

### 5.3. Normal Equation-Based Weighted Moving Average Channel Quality Prediction Algorithm

**Figure 4:**
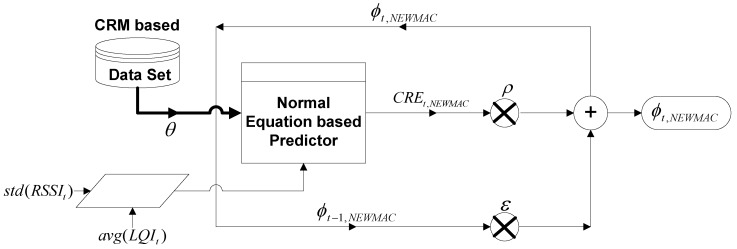
Data flow diagram of NEWMAC algorithm.

The NEWMAC algorithm employs a simple weight moving average-based criterion where the final channel rank estimation is calculated by assigning equal weights to current and past channel quality predictions. Here, current channel quality is predicted by employing the same mechanism as used in the NEC algorithm, whereas the past channel quality is based on the weighted moving average outcome in the previous iteration, as shown in Figure 4. Since the final channel quality assessment is based on past and current channel quality predictions, the NEWMAC algorithm has the ability to accommodate stream-based communication.

#### 5.3.1. Weight Moving Average-Based Channel Quality Prediction Mechanism

Let $\phi_{t-1,NEWMAC}^{i}$ denote the past channel quality prediction and ${\left(CRE\right)}_{t,NEWMAC}^{i}$ denote the current channel rank estimation. Let *ϵ* and *ρ* denote the weights of past and current channel quality predictions, respectively, which have been assigned equal value in this calculation. Then, the moving average-based final channel quality prediction is given by:

(15) $$\phi_{t,NEWMAC}^{i}=\epsilon \;\times \;\phi_{t-1,NEWMAC}^{i}+\rho \;\times \;{\left(CRE\right)}_{t,NEWMAC}^{i}$$

with

(16) ϵ+ρ=1

#### 5.3.2. Problem Definition

Let $\phi_{t,NEWMAC}^{i}$ denote the final quality of any channel *i* at time *t*, being measured by taking the moving average of NEWMAC-based past channel quality prediction and normal equation-based current channel rank estimation. Let *C*, *Z* and *i* be defined as above, again with *C* = *Z* + *i*. Then, the objective function *F* is to select a channel *i* at instant *t* that exhibits the maximum quality:

(17) $$\text{Maximize}:\;\;F(\phi_{t,NEWMAC}^{i})\;,\;\;\;\;\;i=1,2,3\ldots C$$

(18) $$\text{Subject}\;\text{to}:\;\phi_{t,NEWMAC}^{i}\geq \phi_{t,NEWMAC}^{Z}\;,\;\;\;C=\left\{i+Z/i\notin Z\right\}$$

where,

$$0\leq (\phi_{t,NEWMAC}^{i})\leq 1.0$$

The above constraint (Equation (18)) says that the sensor node would select such a channel *i* for stream-based data communication at a particular epoch that exhibits the maximum quality. While the moving average-based NEWMAC algorithm predicts the average channel behavior by considering the current and past channel quality predictions, it is rather slow in tracking channel quality degradations/upgradations at a particular epoch (as discussed in performance evaluation Section 6.1.2), which may adversely affect system performance. Additionally, the NEWMAC algorithm embodies no mechanism for performing channel stability assessment (as discussed in the performance evaluation Section 6.1.3).

### 5.4. Normal Equation-Based Aggregate Maturity Criteria with Beta Tracking Based Channel Weight Prediction Algorithm

**Figure 5:**
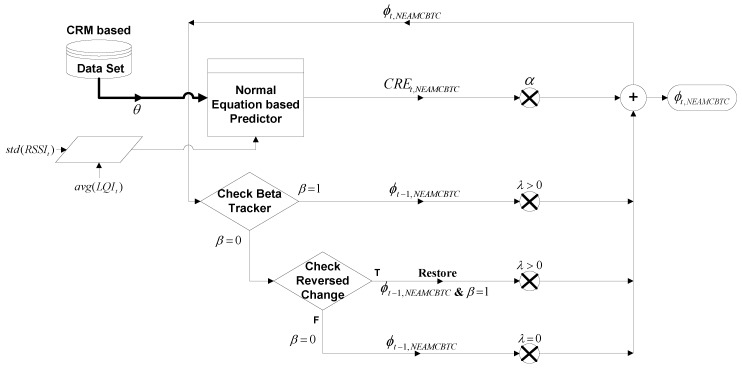
Data flow diagram of NEAMCBTC algorithm.

The NEAMCBTC algorithm has the ability to accommodate stream-based communications, because it estimates the long-term average channel quality on the basis of current and past channel rank estimations, as shown in Figure 5. It employs a dynamic channel maturity criterion as a measure of quality-stability of a channel discussed in Section 5.4.1. Moreover, it has the ability to immediately track any change in channel quality as explained in Section 5.4.2.

Let ${\left(CRE\right)}_{t,NEAMCBTC}^{i}$ and $\phi_{t-1,NEAMCBTC}^{i}$ denote the current and past channel quality predictions of a channel *i*. Let $\eta_{t}^{i}$ and f(c) represent dynamic weights assigned to current and past channel quality assessment, while *β*-tracker is the measure of channel quality tracking. Then, NEAMCBTC-based channel quality assessment is given by:

(19) $$\phi_{t,NEAMCBTC}^{i}=\left(\beta -tracker\right)\;\times \;\left(f(c)\right)\;\times \;\phi_{t-1,NEAMCBTC}^{i}+\left(\frac{1}{\eta_{t}^{i}}\right)\;\times \;{\left(CRE\right)}_{t,NEAMCBTC}^{i}$$

It is clear from Equation (19) that NEAMCBTC-based channel quality assessment is performed with the help of four interconnected procedures, i.e., channel maturity criterion *η*, channel stability criterion *ψ*, channel tracking criterion *β*-tracker and a circular function f(c), as explained below.

#### 5.4.1. Channel Maturity Criterion (*η*)

Unlike the NEWMAC algorithm, the NEAMCBTC algorithm employs a dynamic channel weighting procedure controlled by $\eta_{t}^{i}$ , which adaptively assigns weights to past and current channel quality predictions. If the channel quality level is sustained, then the value of $\eta_{t}^{i}$ is matured (incremented) with time using general-stability criterion $\psi_{t}^{i}$ (Equation (20)) and subsequently increases confidence on past channel quality prediction as determined by circular function f(c), unless either the maximum maturity limit (here $\eta_{t}^{i}=10$ ) is obtained or the channel quality is majorly/minorly degraded/upgraded and the channel maturity procedure is resumed again (here $\eta_{t}^{i}=1$ ). Mathematically, we can write:

(20) $$\eta_{t}^{i}=\left\{ \begin{array}{cc} \psi_{t}^{i}, & \;\text{if}\;\psi_{t}^{i}\leq 10\\ 10, & \;\text{Otherwise} \end{array}\right.$$

and:

(21) $$\lambda_{t}^{i}=f\left(c\right)=\left\{ \begin{array}{cc} \left(\frac{\eta_{t}^{i}-1}{\eta_{t}^{i}}\right), & \;\text{if}\;\eta_{t}^{i}>1\\ \;0\;, & \;\text{Otherwise} \end{array}\right.$$

#### 5.4.2. Channel Tracking Criterion (*β*-*Tracker*)

The *β*-tracker continuously monitors channel quality levels Q(ch) shown in Table 4 and makes the appropriate decision in the case of any degradation/upgradation in channel quality, as outlined in Table 5. As a result, it may adjust the channel stability criterion accordingly (Equation (25)). Moreover, when a channel enters into new quality level, then both *β*-tracker and circular function f(c) nullify the past channel rank estimation $\left(\phi_{t-1,NEAMCBTC}^{i}\right)$ . The NEAMCBTC algorithm also employs a mechanism for handling any abnormal channel quality degradation/improvement, as discussed in Section 6.1.2.

The *β*-tracker is dependent on the current and previous values of channel quality level estimations $Q(ch_{t,t-1}^{i})$ and helps to track any change in channel quality level as calculated below:

(22) $$\beta_{t,NEAMCBTC}^{i}=\langle \frac{MIN\left(Q{\left(ch\right)}_{t,NEAMCBTC}^{i}\;,\;Q{\left(ch\right)}_{t-1,NEAMCBTC}^{i}\right)}{MAX\left(Q{\left(ch\right)}_{t,NEAMCBTC}^{i}\;,\;Q{\left(ch\right)}_{t-1,NEAMCBTC}^{i}\right)}\rangle$$

Rewriting Equation (19) in simplified form, we get:

(23) $$\phi_{t,NEAMCBTC}^{i}=\beta_{t}^{i}\;\times \;\lambda_{t}^{i}\;\times \;\phi_{t-1,NEAMCBTC}^{i}+\alpha_{t}^{i}\;\times \;{\left(CRE\right)}_{t,NEAMCBTC}^{i}$$

with:

(24) $$\alpha_{t}^{i}+\lambda_{t}^{i}=1$$

#### 5.4.3. Channel General-Stability Criterion (*ψ*)

The channel general-stability criterion *ψ* is the measure of time since when a channel resides in a specific quality level (i.e., good/intermediate/bad). In this respect, a channel is considered more stable if it maintains a particular quality level for a prolonged interval. When a channel shifts to a new quality level, then the channel stability criterion *ψ* is resumed and incremented on each interval as long as the channel remains in that particular quality level as shown in the following equation:

(25) $$\psi_{t}^{i}=\left\{ \begin{array}{ll} \psi_{\left(t-1\right)}^{i}+1, & \;\text{if}\;\beta_{t}^{i}=1\\ 1, & \;\text{Otherwise} \end{array}\right.$$

#### 5.4.4. Problem Definition

Since, the behavior of a channel varies with time, the channel quality assessment has to be made repeatedly. For stream-based transmission on a channel, the quality assessment becomes even more critical because it involves sending more volume of data and reserving the channel for a prolonged interval. Therefore, if the channel quality assessment is done appropriately, it results in selecting an appropriate channel for performing future data transmission and routing. As a consequence, higher throughput and better system reliability may be achieved.

Let $\phi_{t,NEAMCBTC}^{i}$ denotes the final quality of any channel *i* at time *t*, which is determined through NEAMCBTC-based past channel quality prediction and normal equation-based current channel rank estimation. Let *C*, *Z* and *i* be defined as above with *C* = *Z* + *i*. Then, the objective function *F* is to select a channel *i* at instant *t* that exhibits maximum quality:

(26) $$\text{Maximize}:\;\;F(\phi_{t,NEAMCBTC}^{i})\;,\;\;\;\;\;i=1,2,3\ldots C$$

(27) $$\text{Subject}\;\text{to}:\;\phi_{t,NEAMCBTC}^{i}\geq \phi_{t,NEAMCBTC}^{Z}\;,\;\;C=\left\{i+Z/i\notin Z\right\}$$

where,

$$0\leq (\phi_{t,NEAMCBTC}^{i})\leq 1.0$$

The above constraint (Equation (27)) states that the channel *i* with the highest quality will be selected for performing stream-based data communication at instant *t*. Since the NEAMCBTC algorithm also employs the channel maturity criterion (*η*) and the channel tracking criterion (*β*-tracker), therefore it has the capability to perform limited quality stability and to track instantaneously any major/minor channel in channel quality, respectively. That is why the NEAMCBTC algorithm is more suitable to accommodate stream-based data communication than the NEWMAC algorithm.

#### 5.4.5. Extended-NEAMCBTC Algorithm (with General Stability Assessment)

When channel quality is majorly/minorly degraded/upgraded, then the NEAMCBTC algorithm resumes the channel maturity criterion from scratch. This may result in preferring a channel that has just attained the best quality, although it may have suffered from instability in the recent past (e.g., see the behavior of Channel 7 between interval [36, 39], as discussed in Section 6.1.1). This issue can be handled if we consider the general stability criterion $\psi_{t}^{i}$ as a metric in the final channel quality estimation $\phi_{t,NEAMCBTC}^{i}$ of any channel *i*. The resulting metric ( $\xi_{t,Ext-NEAMCBTC}^{i}$ ) would be more robust as given below:

(28) $$\xi_{t,Ext-NEAMCBTC}^{i}=\phi_{t,NEAMCBTC}^{i}+\psi_{t}^{i}$$

Rewriting Equation (23), we get:

(29) $$\xi_{t,Ext-NEAMCBTC}^{i}=\left(\beta_{t}^{i}\;\times \;\lambda_{t}^{i}\;\times \;\phi_{t-1,NEAMCBTC}^{i}+\alpha_{t}^{i}\;\times \;{\left(CRE\right)}_{t,NEAMCBTC}^{i}\right)+\psi_{t}^{i}$$

**Figure 6:**
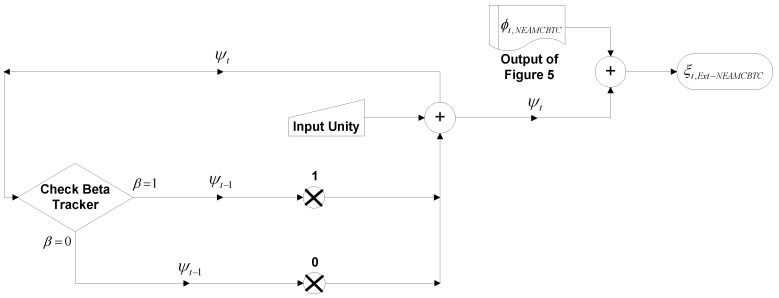
Data flow diagram of the Ext-NEAMCBTC algorithm.

The $\xi_{t}^{i}$ metric may enable sensor nodes to predict both quality and stability and make a better choice among available channels for performing stream-based communications. The data flow diagram of the Ext-NEAMCBTC algorithm is shown in Figure 6.

## 6. Performance Evaluation

For measuring the performance of the proposed machine learning-based algorithms, we have conducted extensive simulations in MATLAB. In this respect, the experimental section can be divided into two main sub-sections:

- In the first portion of simulations, we discuss channel quality and stability assessment of our machine learning-based algorithms on the basis of randomly-generated samples of $std(RSSI_{t}^{i})$ and $avg(LQI_{t}^{i})$ in various channel quality ranges, as shown in Table 6, and representing the quality of seven channels. For this purpose, we have assumed that sensor nodes have some inherent mechanism for calculating $std(RSSI_{t}^{i})$ and $avg(LQI_{t}^{i})$ on the basis of received packets. The concluding remarks of this section are outlined in Section 6.1.4 and Table 7.
- In the second portion of simulations, we perform channel switching energy $Energy_{Ch-Switch}$ and channel switching delay $Delay_{Ch-Switch}$ related measurements of all discussed algorithms and compare them accordingly. Since, to the best of our knowledge, there is no multichannel scheme for WSNs similar to our work, therefore we have compared the performance of our schemes to the following approaches.
  i Random selfish approach: In this technique, the channel is selected randomly among all of the available channels. Afterwards, the sensor node communicates on the selected channel as long as the channel quality is either good $\left(Ch_{good}\right)$ or intermediate $\left(Ch_{inter}\right)$ .
  ii EM-MAC-based approach: It follows the pseudo-random order-based frequency hopping mechanism of EM-MAC [26], whereby channels of acceptable quality are hopped only in a pseudo-random manner, while bad quality channels $\left(Ch_{bad}\right)$ , once identified, are marked as blacklisted for a specific time interval.

### 6.1. Channel Quality and Stability Assessment Using the Proposed Machine Learning-Based Algorithms

In this section, we simulate and discuss the functionality of proposed algorithms for accommodating stream-based communication. For this purpose, the proposed algorithms are evaluated using three main factors as given below.

#### 6.1.1. Channel Quality Assessment

**Figure 7:**
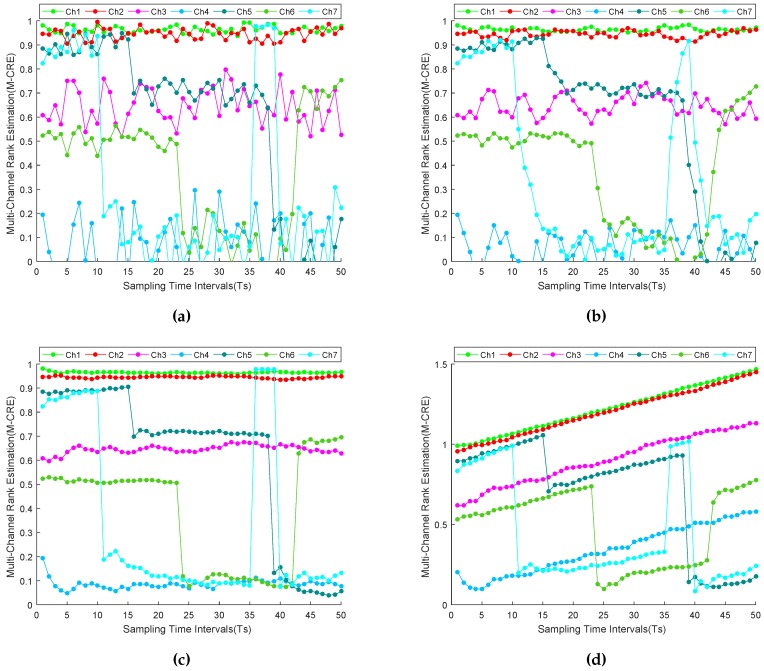
Channel quality (and stability) assessment results. (**a**) results for NEC; (**b**) results for NEWMAC; (**c**) results for NEAMCBTC; (**d**) results for Ext-NEAMCBTC (with stability).

Stream-based communication requires transmitting chunks of information from source to destination rather than performing packet-by-packet delivery of data. When the quality of available channels is overlapping, then those channel quality assessment (CQA) approaches that perform channel quality estimation on the basis of only instantaneous observation(s) of channel quality may suffer from frequent channel switching overheads. This is due to the fact that the sensor node may tend to occupy the best quality channel at each epoch, which may result in inducing frequent channel switchings at the corresponding epochs. Such frequent channel switching may be risky and, therefore, is avoided for high data-rate applications, such as stream-based communication, because it may result in additional energy consumption and data loss [12]. This can be seen, for instance, in Figure 7a, where an NEC algorithm-based sensor node mostly switches between Channels 1 and 2 and sometimes to Channel 7. In addition, at Time Instances 7, 19 and 33, the sensor node rapidly shifts between Channels 1 and 2, which increases the channel switching overhead. Therefore, the NEC algorithm is unable to perform channel quality assessment required for stream-based communication.

Figure 7b shows the results for NEWMAC algorithm. Here, channel switching happens only between Channels 1 and 2 at Time Instances 12, 20, 30, 46 and 48, which may induce channel switching overhead in stream-based communication. In contrast, the NEAMCBTC algorithm increases the confidence on past channel rank estimation with time. As a result, it gives a better estimate of long-term average channel quality and handles any short-term channel quality degradations and upgradations. From Figure 7c, it is obvious that the NEAMCBTC algorithm clearly estimates the superiority of Channel 1 over Channel 2; therefore, no switching happens. Additionally, it suffers from only one round-trip channel switching overhead between Channels 1 and 7, and therefore, NEAMCBTC algorithm is more suitable to accommodate stream-based communication in WSNs than NEWMAC algorithm. From Figure 7d, it is evident that Ext-NEAMCBTC algorithm handles the round-trip channel switching overhead between Channels 1 and 7 in the Time Interval [36, 39], therefore Ext-NEAMCBTC algorithm performs better than the other devised algorithms.

#### 6.1.2. Channel Quality-Tracking Assessment

Channel quality-tracking assessment (CQTA) is the measure of an algorithm’s ability to promptly pursue any minor/major degradation/upgradation in channel quality. As shown in Table 5, minor degradation or upgradation happens when the quality of a channel changes from a higher to an adjacent lower level, or vice versa, whereas major degradation or upgradation in channel quality takes place when channel quality decreases from good to bad level, or vice versa. The CQTA of our algorithms is discussed below.

**Figure 8:**
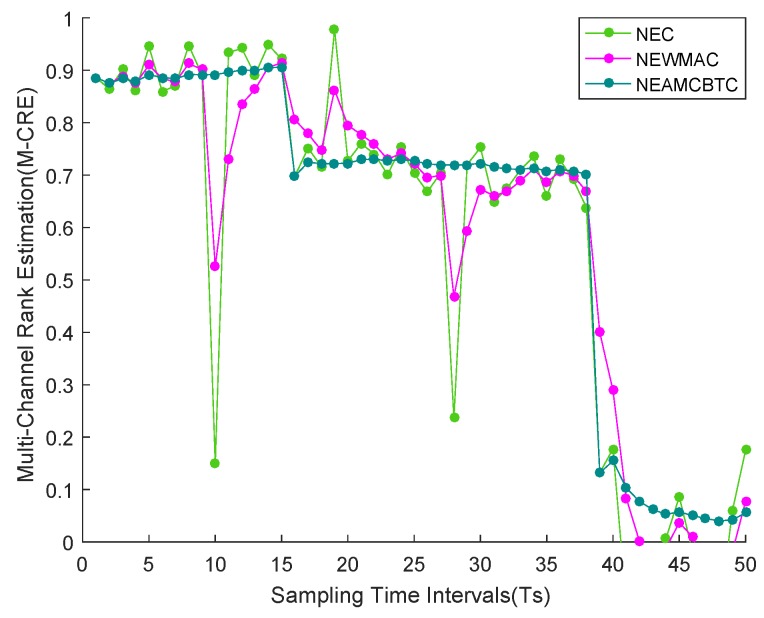
Channel abnormal behavior tracking and healing.

- NEC-based channel tracking: The NEC algorithm performs channel rank estimation on the basis of instantaneous channel quality observations(s); therefore, it provides prompt knowledge of a channel quality change, as shown in Figure 7a.
- NEWMAC-based channel tracking: Due to its moving average-based design, NEWMAC is unable to quickly respond to any change in channel quality. For example, looking at instantaneous knowledge based Figure 7a at Time Instant 11, Channel 7 is jammed. However, due to slow tracking ability, the NEWMAC algorithm still considers Channel 7 as of intermediate quality at Instant 11 and therefore prefers Channel 7 over Channel 6, as shown in Figure 7b. Consequently, this may result in system performance degradation. Similarly, when Channel 7 recovers from jamming at Time Instant 36, then the NEWMAC algorithm again takes some time in tracking the new quality of Channel 7. Hence, due to its poor tracking ability, the NEWMAC algorithm is not a good candidate for performing stream-based communication.
- NEAMCBTC-based channel tracking: Whenever any meaningful change in channel quality level is observed, then it is immediately tracked by *β*-tracker, which equalizes its channel quality tracking ability to that of NEC algorithm, as shown in Figure 7c. For example, as soon as Channel 7 suffers from jamming attack at Time Instant 11, then NEAMCBTC immediately tracks it, as shown in Figure 7c, and thereby, avoids Channel 7. Unlike NEWMAC, NEAMCBTC is able to promptly track recovery of Channel 7 from jamming, as shown in Figure 7c, which makes it a good candidate for accommodating stream-based communication. Since Ext-NEAMCBTC is based on NEAMCBTC, therefore it can promptly track any major/minor degradation/upgradation in channel quality at a particular epoch and can accommodate stream-based communication in WSNs.
- Channel abnormal behavior tracking and healing: Sometimes, instantaneous distortions in channel quality estimations crop up due to environmental factors, which may strongly effect the prediction capability of those memory-based systems that consider past knowledge in the future channel quality estimations. Unlike NEWMAC, NEAMCBTC has the ability to effectively handle any such instantaneous quality distortions. This capability of NEAMCBTC helps its channel maturity criterion to function properly and make better decisions for final channel rank estimations. For example, we have deliberately introduced short-term minor/major abnormalities in Channel 5 and drawn its graph using the NEC, NEWMAC and NEAMCBTC algorithms, as shown in Figure 8. Being instantaneous knowledge based, NEC is not affected by any such irregularities from the past. On the other hand, NEWMAC is strongly affected by those oddnesses, while NEAMCBTC employs an inherent mechanism for suppressing these instantaneous abnormal distortions.

#### 6.1.3. Channel Stability Assessment

The stability of a channel is the measure of time during which a channel occupies a particular channel quality level. When stability is also considered as a metric for determining the channel rank, then good quality stable channels are assigned more weight and preferred over good quality unstable channels for performing stream-based communication.

More specifically, channel quality assessment aims to select the best quality channel, whereas channel stability assessment focuses on selecting a channel whose quality may remain steady for a prolonged interval. The above discussion has realized the fact that although NEAMCBTC is superior to its counterparts; it embodies only partial quality stability. Therefore, it handles both good quality stable/unstable channels using a similar mechanism, as shown for instance in Figure 7c, where good quality unstable Channel 7 is preferred over good quality stable Channel 1 in the time interval [36, 39]. To bridge this gap, the extension Ext-NEAMCBTC incorporates a composite metric (Equation (29)), which considers both channel quality and stability. It enables sensor nodes to prefer Channels 1, 2 and 3 over Channel 7 for time interval [36, 39], as shown in Figure 7d, and thus, enhances the capability of our system to accommodate stream-based communication.

#### 6.1.4. Concluding Remarks: A Brief Discussion

In this section, we will summarize the pros and cons of our algorithms and their appropriateness for performing stream-based data communication in WSNs, as outlined in Table 7.

Since NEC makes estimations on the basis of current channel quality observation(s) only, therefore it is computationally the lightest among the discussed algorithms and can figure out the best among the available channels at the current epoch, which enables it to perform channel quality tracking easily. On the other hand, it is unable to give any long-term/average prediction of the channel quality and may suffer from frequent channel switching overheads, which makes it a poor choice for accommodating stream-based communication.

The moving average-based NEWMAC algorithm estimates channel quality on the basis of both past channel knowledge and current channel quality observation(s). Thus, it can predict the average behavior of a channel and may avoid frequent channel switching overheads, which makes it a better candidate for performing stream-based communication. Being based on the moving average approach, it requires more memory and processing power than NEC and exhibits a slow growth rate, which is unsuitable for promptly tracking any major/minor changes in channel quality. Hence, in an environment where channels are suffering from rapid quality degradations and/or upgradations, NEWMAC may not provide suitable knowledge of channel quality for accommodating stream-based communication.

NEAMCBTC, finally, estimates channel quality using an adaptive channel maturity criterion that dynamically assigns weight to past channel knowledge and current channel rank observations(s). Thus, it gets long-term average channel behavior required for handling channel switching overheads and accommodating stream-based communication. The approach also embodies a robust channel tracking mechanism, which may accurately track any major/minor change in channel quality. Due to increased functionality, the algorithm may require more memory and processing capability as compared to the already discussed algorithms.

The NEAMCBTC algorithm, however, suffers from preferring those unstable channels that may exhibit better quality than stable channels, even for short intervals of time. This may result in inducing limited channel switching overheads, which may limit the performance of NEAMCBTC for accommodating stream-based communication. The extension, Ext-NEAMCBTC algorithm, solves this outstanding issue and encourages justice between stable channels and good quality unstable channels.

### 6.2. Measurement of Channel Switching Overhead

This section discusses channel switching overheads in terms of switching delay and energy consumption of relevant algorithms, which may help to figure our their efficiency and suitability for accommodating stream-based communication. For more realistic calculations, we have utilized channel switching delay and energy consumption values outlined in [34].

#### 6.2.1. Channel Switching Energy Overhead

Channel switching energy overhead is the amount of energy consumed by a sensor node when it jumps from one channel to another. The total energy consumed in channel switching is the sum of the energy consumed in calibrating (RX,TX) and restarting the radio afterwards [34]:

(30) $$Energy_{Ch-Switch}=\left[Energy_{Cb_{RX}}\right]+\left[Energy_{Cb_{TX}}\right]+\left[Energy_{T_{restart}}\right]$$

**Figure 9:**
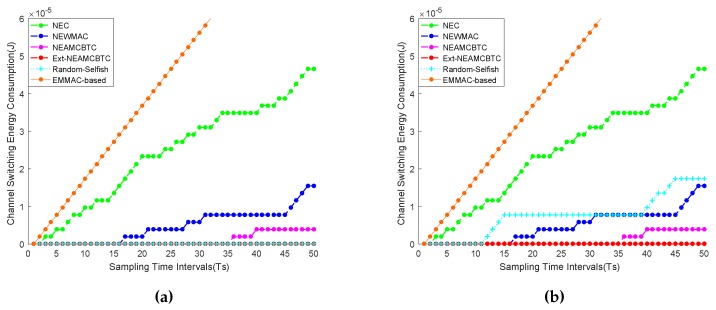
Channel switching energy overhead. (**a**) Case I: the channel switching energy consumption of the compared techniques shows that Ext-NEAMCBTC and random selfish (in the above scenario) are the best due to no switching energy overhead, while the EM-MAC-based approach behaves the worst among all. (**b**) Case II: the channel switching energy consumption of the compared techniques shows that Ext-NEAMCBTC is superior while random selfish (in the above scenario) performs worse than the NEWMAC approach. Random channel selection gives varying behavior to the random selfish approach.

It is clear from the Figure 9a,b that Ext-NEAMCBTC performs better as compared to other algorithms because it allows sensor nodes to jump to the channel of best quality and stability. Moreover, the sensor node resides on the best available channel as long as the quality of that channel is better than the other available channels. Since Ext-NEAMCBTC helps the sensor node to minimize channel switching overhead as much as possible by taking preemptive channel assessment measures, it encounters the least channel switching energy consumption among the compared techniques. On the other hand, the performance of the EM-MAC-based approach is the lowest among all available approaches because it follows a procedure with frequent frequency hopping, which allows it to switch from one channel to another.

Additionally, the random selfish approach consumes 0–1.8 × 10^−5^ J energy by making 0–9 channel switchings, respectively, in the current scenario, as is evident from Figure 9a,b. This is due to the fact that the random selfish approach hops channels randomly and stays on a channel as long as the channel quality is good. Therefore, if it randomly hops to a channel of good quality and stability, it suffers from the least channel switching overhead, as shown in Figure 9a. On the other hand, if the random selfish approach selects channels that are either of bad quality or their quality is degraded soon, then it may suffer from frequent channel switchings, resulting in an increase in channel switching energy budget, as shown in Figure 9b. Consequently, such random behavior may cause performance issues, which may be considered preemptively while employing random-based approaches.

#### 6.2.2. Channel Switching Delay Overhead

The switching delay is the summation of latency experienced by a sensor node while shifting from one channel to other. It is the measure of delay experienced in calibrating (RX,TX) and restarting the radio afterwards [34]. Mathematically, we can write:

(31) $$Delay_{Ch-Switch}=\left[Delay_{Cb_{RX}}\right]+\left[Delay_{Cb_{TX}}\right]+\left[Delay_{T_{restart}}\right]$$

**Figure 10:**
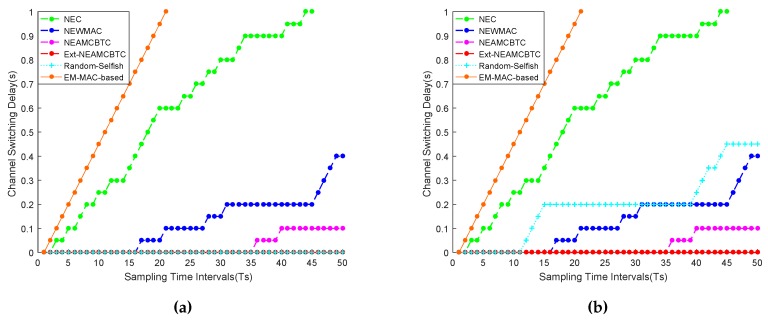
Channel switching delay overhead. (**a**) Case I: the channel switching delay measurement of discussed algorithms where Ext-NEAMCBTC and random selfish (in the above scenario) are behaving the best owing to no switching delay overhead, whereas the EM-MAC-based approach behaves the worst due to frequent channel hopping; (**b**) Case II: the channel switching delay measurement of discussed algorithms where Ext-NEAMCBTC behaves the best while random selfish (in the above scenario) behaves worse than NEWMAC technique. The varying behavior of random selfish approach is due to random channel selection.

It is clear from Figure 10a,b that Ext-NEAMCBTC suffers from the least channel switching delay among the compared approaches because it has the ability to categorize channels on the basis of its inherent channel quality and stability assessment mechanism. As a result, it avoids channel switching delays as much as possible, which may result in timely delivery of data-stream and ensuring system reliability through avoiding switching-oriented data losses. On the other hand, frequent channel hopping in EM-MAC-based may cause the most persistent channel switching delays and data losses, which makes it a poor candidate for accommodating stream-based communication.

Moreover, the random selfish-based approach shows a mixed trend, as evidenced from Figure 10a,b, whereby it may either perform as good as Ext-NEAMCBTC in case it randomly occupies a channel of good quality and stability or it may perform worse than the NEWMAC algorithm in case it is unable to randomly hop to channels of good quality and stability. Thus, the random selfish technique may suffer from a channel switching delay between 0 and 0.45 s owing to 0–9 channel switchings, respectively, in the current scenario, as evidenced from Figure 10a,b.

## 7. Conclusions

Conventional high data rate multichannel applications may perform per-packet handshaking, which may result in channel switching overheads and data losses. The solution is to perform stream-based communication that may require channel handshaking before transmitting the whole data stream and, consequently, handle channel switching overheads while ensuring network reliability. Efficient stream-based communication is possible when the behavior of channels is known before transmitting the whole data stream. For effective realization of channel behavior, it is important to consider both channel quality and stability because it results in making better decisions for overall channel quality assessment in stream-based multichannel wireless sensor networks and may increase network efficiency and reliability by avoiding unnecessary channel switchings. However, selecting a channel of good quality and stability is a challenging task. In this work, an initiative is taken for devising, comparing and analyzing different techniques in order to figure out the best among them that can perform both channel quality and stability assessment and assist sensor nodes in selecting the best among all available channels for performing stream-based communication and thereby achieving high performance in high data rate multichannel wireless sensor networks. In this way, channel switching overheads, such as switching delays, energy consumption and data losses, would be minimized, and network efficiency is ensured accordingly.

## Figures and Tables

**Table 1:** Demarcation link types. LQI, link quality indicator.

Link Type	*std*(*RSSI*)	*avg*(*LQI*)
Good	<4	>104
Intermediate	4–10	70 to ≤104
Bad	>10	<70

**Table 2:** Summary of related protocols reviewed. ETX, expected number of transmissions.

Protocol	Field	Current Knowledge	Past Knowledge
RMCA [30]	Multichannel	−	Regret matching based
EM-MAC [26]	Multichannel	Interference based	−
DRCS [12]	Multichannel routing	Battery power and ETX based	−
[29]	Multichannel routing	−	Game-theory based
MMOCR [28]	Multichannel Routing	RSSI and SINR based	−

**Table 3:** Channel rank measurement metric.

Channel Type	*std*(*RSSI*)	*avg*(*LQI*)	*scale*(*RSSI*)	*scale*(*LQI*)	*CRM*
Good	<4	>104	11< to ≤15	13.52 to 15	0.82≤ to ≤1.00
Intermediate	4–10	70≤ to ≤104	5 to 11	5 to 13.50	0.33≤ to <0.82
Bad	>10	<70	0≤ to <5	0 to 4.97	0≤ to <0.33

**Table 4:** *β*- tracker based channel quality level Q(ch) assignment with $q_{1}=0.3$ , $q_{2}=0.2$ , $q_{3}=0.1$ .

Serial No.	Channel Type	*CRE*	*Q*(*ch*)
1.	Good	0.82≤ to ≤1.00	$q_{1}$
2.	Intermediate	0.33≤ to <0.82	$q_{2}$
3.	Bad	0≤ to <0.33	$q_{3}$

**Table 5:** *β*- tracker -based channel decision making.

Serial No.	$Q({\mathrm{ch}}_{t-1}^{i})$	$Q({\mathrm{ch}}_{t}^{i})$	$\beta_{t}^{i}$ -*Tracker* Decision Making	Channel Quality Explanation
1	$q_{1}$	$q_{1}$	1	Maintaining Good Quality
2	$q_{2}$	$q_{2}$	1	Maintaining Intermediate Quality
3	$q_{3}$	$q_{3}$	1	Maintaining Bad Quality
4	$q_{1}$	$q_{2}$	0	Minor Change (to Intermediate Quality)
5	$q_{2}$	$q_{1}$	0	Minor Change (to Good Quality)
6	$q_{2}$	$q_{3}$	0	Minor Change (to Low Quality)
7	$q_{3}$	$q_{2}$	0	Minor Change (to Intermediate Quality)
8	$q_{1}$	$q_{3}$	0	Major Change (to Bad Quality)
9	$q_{3}$	$q_{1}$	0	Major Change (to Good Quality)

**Table 6:** Simulation parameters.

Symbol	Description	Value
*N*	Number of channels	7
$\theta_{o}$	Machine learning based weight of parameter $X_{0}$	0.0824
$\theta_{1}$	Machine learning based weight of parameter $X_{1}$	−0.0333
$\theta_{2}$	Machine learning based weight of parameter $X_{2}$	0.0083
$std{\left(RSSI\right)}_{good}$	Standard deviation RSSI of good quality channel	<4 [20]
$avg{\left(LQI\right)}_{good}$	Average LQI of good quality channel	>104 [20]
$std{\left(RSSI\right)}_{inter}$	Standard deviation RSSI of intermediate quality channel	4–10 [20]
$avg{\left(LQI\right)}_{inter}$	Average LQI of intermediate quality channel	70 to ≤104 [20]
$std{\left(RSSI\right)}_{bad}$	Standard deviation RSSI of bad quality channel	>10 [20]
$avg{\left(LQI\right)}_{bad}$	Average LQI of bad quality channel	<70 [20]
$Ch_{good}$	Quality range of good rank channel	0.82≤ to ≤1.00
$Ch_{inter}$	Quality range of intermediate rank channel	0.33≤ to <0.82
$Ch_{bad}$	Quality range of bad rank channel	0.0≤ to <0.33
$T_{s}$	Sampling Time Interval	1 × 10^2^ ms
$Delay_{Ch-Switch}$	Overall channel switching delay	50 ms (approx) [34]
$Delay_{Cb_{RX}}$	Delay in calibrating receiver	22.08 ms [34]
$Delay_{Cb_{TX}}$	Delay in calibrating transmitter	23.44 ms [34]
$Delay_{T_{restart}}$	Delay in restarting radio after calibration	4.32 ms [34]
$Energy_{Ch-Switch}$	Total energy consumption in channel switching	1940 nJ (approx) [34]
$Energy_{Cb_{RX}}$	Energy consumption for calibrating receiver	1005.05952 nJ [34]
$Energy_{Cb_{TX}}$	Energy consumption for calibrating transmitter	838.42536 nJ [34]
$Energy_{T_{restart}}$	Energy consumption for restarting radio after calibration	96.95376 nJ [34]

**Table 7:** Feasibility of the proposed schemes for stream-based communication in multichannel WSNs. CQA, Channel Quality Assessment; CQTA, channel quality-tracking assessment; CSA, channel stability assessment.

Protocol	CQA	CQTA	CSA
NEC	−	✔	−
NEWMAC	✔	−	−
NEAMCBTC	✔	✔	Partial
Ext-NEAMCBTC	✔	✔	✔
